# Experimental infection of Bama miniature pigs with a highly virulent classical swine fever virus

**DOI:** 10.1186/1743-422X-8-452

**Published:** 2011-09-25

**Authors:** Yuan Sun, Qian Jiang, Da-Yong Tian, Huan Lin, Hong Li, Qiu-Ying Han, Wen Han, Chang-De Si, Shou-Ping Hu, Zhuo Zhang, Lian-Dong Qu, Hua-Ji Qiu

**Affiliations:** 1 State Key Laboratory of Veterinary Biotechnology, Harbin Veterinary Research Institute, Chinese Academy of Agricultural Sciences, Harbin 150001, China

**Keywords:** Bama miniature pigs, classical swine fever virus, infection model

## Abstract

**Background:**

Currently, larger domestic pigs are only animals widely used in vaccine evaluation and pathogenicity study of classical swine fever virus (CSFV). This study was aimed to create an alternative animal experimental infection model of CSFV.

**Results:**

Twenty specific-pathogen-free Bama miniature pigs were randomly divided into two groups and rooms, infected and non-infected, and the pigs in the infected group were inoculated intramuscularly with 10^4^, 10^5 ^or 10^6 ^TCID_50 _(median tissue culture infective dose) CSFV Shimen strain (*n *= 5 × 3) or left uninoculated to serve as in-contact pigs (*n *= 3). The uninfected control pigs (*n *= 2) were housed in a separate room. Clinical signs, body temperature, viraemia, tissue antigen distribution, pathological changes and seroconversion were monitored. Clinical signs were observed as early as 2 days post-inoculation (dpi) in all infected pigs (though mild in contact pigs), but not non-infected control pigs. All inoculated pigs showed viraemia by 6 dpi. The in-contact pigs showed lower levels of viraemia. At 10 dpi, seroconversion was noted in five of the 15 inoculated pigs. All inoculated or one in-contact pigs died by 15 dpi.

**Conclusions:**

These results show that Bama miniature pigs support productive CSFV infection and display clinical signs and pathological changes consistent with CSFV infections observed in larger domestic pigs.

## Background

Classical swine fever (CSF) is caused by classical swine fever virus (CSFV) and results in significant losses to the pig industry worldwide. CSFV belongs to the *Pestivirus *genus within the *Flaviviridae *family [1]. It is an enveloped virus containing a single-stranded, positive-sense RNA encoding a 3,898 amino acid polyprotein, which undergoes co- and post-translational processing by cellular and viral proteases to yield 11-12 cleavage products [2,3].

Pigs are the natural host of CSFV, and are used as models for CSFV research. Therefore, vaccines against CSF should be evaluated exclusively in pigs in preclinical and clinical trials. A major challenge, however, is that domestic pigs are large and difficult to handle; thus, a convenient animal model is required for the study of CSF and other swine diseases. Several minipig strains, such as Göttingen, CLAWN, Yucatan, Lanyu, Bama, Sinclair and Hanford, have been used as toxicological and pharmacological models. Minipigs have also been used as a model for experimental infections for some pathogens including *Escherichia coli *[4], *Streptococcus suis *[5], *Schistosoma japonicum *[6], and dengue virus [7]. Chinese Bama miniature pigs are genetically stable, highly inbred, and small (adult mean body weight, 40 kg) [8-10]. The animals are easy to handle compared to larger domestic pigs. In addition, it is feasible to take repeated samples of sufficient volume to enable vaccine studies. This makes the breed an excellent model for use in study on cardiovascular and gastrointestinal diseases, *Helicobacter pylori *infection, renal disease, skin pharmacology, and xenotransplantation [11,12]. The small size of the animals makes them ideal an infection model and an attractive alternative to larger domestic pigs, especially for long-term trials. Recently, specific-pathogen-free (SPF) Bama miniature pig populations have been established in China as experimental animals for medical and veterinary applications.

To the best of our knowledge, there are no published reports on Bama miniature pigs experimentally infected with CSFV. This study details the results of experiments in which Bama miniature pigs were experimentally infected with the highly virulent Shimen strain of CSFV.

## Results

### Clinical features of experimentally-infected Bama miniature pigs

**Figure 1:**
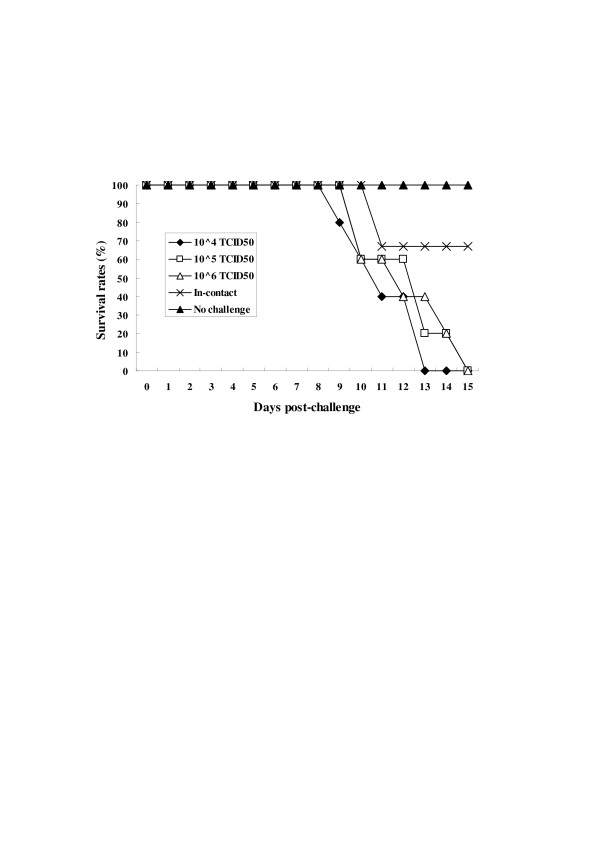
**Survival rates of pigs challenged with different doses of CSFV**. Groups A, B and C (*n *= 5) were inoculated i.m. with 10^4^, 10^5 ^and 10^6 ^TCID_50 _CSFV Shimen strain, respectively. Three non-inoculated pigs were housed in the same pen and served as in-contact animals. Two uninfected control pigs were housed in a separate pen.

Previous studies showed that domestic pigs challenged with 10^5 ^TCID_50 _CSFV Shimen strain exhibited severe clinical signs typical of CSF [13]. Therefore, in the present study, Bama miniature pigs were inoculated i.m. with different doses of CSFV (10^4^, 10^5 ^or 10^6 ^TCID_50_). The results showed that all pigs in groups A (infected with 10^4 ^TCID_50_), B (10^5 ^TCID_50_), and C (10^6 ^TCID_50_) showed clinical signs (fever, shivering and anorexia) at 2 (groups A and B) and 3 (group C) days post-inoculation (dpi), accompanied by a significant increase in rectal temperature. The clinical outcomes for each pig following viral challenge are summarised in Table 1. The incidence of fever was significantly higher in group A than in group C (*P *< 0.05); however, no obvious difference was observed between groups A and B. These early clinical signs were followed by loss of appetite, lethargy, stiffness of gait, reddening of the conjunctiva and loose stools or diarrhoea. As the disease progressed the clinical scores were > 6, and the pigs began to die at 13 dpi. Notably, no reddening, haemorrhage, or petechiae were observed in the three in-contact animals before 10 dpi. These in-contact pigs initially displayed mild clinical signs at 10 dpi. Surprisingly, the clinical scores were very low prior to death. Death occurred from 9 dpi in groups A, B, and C. At 15 dpi, the survival rates in groups A, B, C, D and E were 0/5, 0/5, 0/5, 2/3 and 2/2, respectively (Figure 1). The control pigs in group E remained healthy throughout the trial.

**Table 1:** Clinical outcome of the pigs following viral challenge.

Groups	No. of animals with fever (≥ 40.5°C)	No. of days to fever onset	Fever frequency during the trial	Survival rates
A	5/5	2	26/56	0/5
B	5/5	2	21/51	0/5
C	5/5	3	18/58	0/5
D	3/3	10	3/11	2/3
E	0/2	-	0/30	2/2

Twenty specific-pathogen-free Bama miniature pigs were randomly divided into five groups. Groups A, B and C (*n *= 5) were inoculated i.m. with 10^4^, 10^5 ^and 10^6 ^TCID_50 _CSFV Shimen strain, respectively. Three non-inoculated pigs were housed in the same pen and served as in-contact animals. Two uninfected control pigs were housed in a separate pen. Clinical outcome, fever frequency and survival rates after viral challenge were calculated.

### Antibody responses to CSFV infection

At 7 dpi, no anti-CSFV antibodies were detectable in any of the pigs. At 10 dpi, the pigs began to seroconvert: one in group A (antibody blocking rate, 43.7%), one in group B (45%) and two in group C (51.3% and 64%). At 15 dpi, one more pig in group B seroconverted (43.5%).

### Viraemia and viral RNA distribution in the tissues of experimentally infected pigs

**Figure 2:**
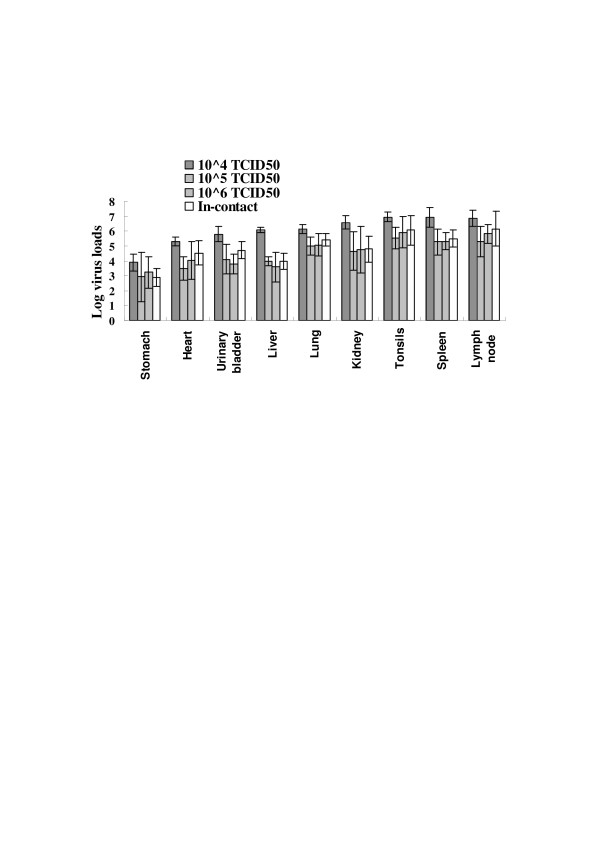
**Log number of viral RNA copies from challenged pigs quantified using real-time RT-PCR (copies/μL)**. Heart, liver, spleen, lung, kidney, stomach, urinary bladder, tonsils and lymph nodes were collected and the distributions of CSFV RNA determined.

Serum samples collected prior to inoculation and at 0, 1, 3, 7, 10 and 15 dpi and tissues collected at necropsy were tested for viral RNA using the real-time RT-PCR as described previously. Viral RNA was detected in the serum of all pigs (except non-infected controls) at 3 dpi, with the viral loads peaking between 7 to 10 dpi (Table 2). Animals inoculated with 10^6 ^TCID_50 _CSFV Shimen strain developed early and severe clinical signs and showed higher viral RNA loads (up to 10^6 ^copies/μL) than the other inoculated animals. The in-contact pigs developed fewer clinical signs and became viraemic 10 dpi. The viral RNA load was < 10^5 ^copies/μL (Table 2). The CSFV RNA load in the different tissues was also examined. Generally, the viral load was higher in pigs challenged with 10^4 ^TCID_50 _than in the other pigs. Higher level viral RNA was detected in the lymph nodes, tonsils, spleen, and lung than in the urinary bladder and stomach (Figure 2). The level of viral RNA in the tissues of pigs challenged with 10^4 ^TCID_50 _CSFV was significantly higher than that in pigs challenged with 10^5 ^TCID_50 _or 10^6 ^TCID_50 _or in-contact pigs (*P *< 0.01). There was no difference between groups B, C and D (Figure 2). No viral RNA was detected in the two uninfected control pigs.

**Table 2:** Detection of viral RNA in serum samples from challenged pigs by real-time RT-PCR (copies/μL).

No.	Days post-challenge					
	
	0	1	3	7	10	15
A1	-	-	2.51 × 10^3^	1.44 × 10^4^	1.78 × 10^4^	/
A2	-	-	1.69 × 10^4^	8.50 × 10^3^	2.47 × 10^4^	/
A3	-	-	8.19 × 10^3^	1.12 × 10^4^	1.06 × 10^4^	/
A4	-	-	1.15 × 10^4^	2.56 × 10^4^	1.33 × 10^4^	/
A5	-	-	7.45 × 10^3^	5.55 × 10^4^	/	/

B1	-	-	2.22 × 10^5^	6.60 × 10^4^	7.05 × 10^5^	/
B2	-	-	6.01 × 10^4^	3.70 × 10^5^	2.38 × 10^5^	/
B3	-	-	1.94 × 10^5^	1.44 × 10^5^	5.23 × 10^5^	6.70 × 10^5^
B4	-	-	1.10 × 10^5^	1.43 × 10^5^	/	/
B5	-	-	1.19 × 10^5^	9.35 × 10^4^	3.46 × 10^5^	/

C1	-	-	6.55 × 10^5^	2.21 × 10^6^	/	/
C2	-	-	7.56 × 10^5^	9.57 × 10^5^	1.49 × 10^6^	/
C3	-	-	1.16 × 10^6^	1.23 × 10^6^	1.06 × 10^6^	2.31 × 10^6^
C4	-	-	4.24 × 10^5^	1.13 × 10^6^	2.63 × 10^6^	/
C5	-	-	3.95 × 10^5^	1.44 × 10^4^	/	/

D1	-	-	3.81 × 10^4^	1.35 × 10^4^	7.79 × 10^5^	5.40 × 10^5^
D2	-	-	2.65 × 10^3^	6.67 × 10^3^	3.07 × 10^4^	/
D3	-	-	3.98 × 10^3^	8.05 × 10^5^	2.21 × 10^4^	5.53 × 10^5^

E1	-	-	-	-	-	-
E2	-	-	-	-	-	-

Serum samples were collected prior to inoculation and at 1, 3, 7, 10 and 15 dpi and tested for viral RNA using the real-time RT-PCR as described previously (Zhao et al., 2008).

### Pathological and histopathological findings

Pigs died of infection were, subjected to pathological and histopathological examination following standard operational procedures, and any observed lesions recorded. All the surviving pigs were euthanised at 15 dpi and also subjected to pathological and histopathological examination. Inoculated pigs showed lesions typical of CSF, such as pinpoint haemorrhaging in the kidney or haemorrhaging within the lymph nodes and urinary bladder, necrosis in the tonsils, and splenic infarcts. Such lesions are typical in larger domestic pigs infected with CSFV. There were also obvious lesions in the liver and lungs and the stomach showed large areas of haemorrhage and ulcers. Although no obvious clinical signs were observed in the in-contact pigs, there was evidence of specific CSF-induced pathological changes. The scores for the pathological changes in each group are shown in Table 3. Infected pigs also showed severe haemorrhaging within the stomach, intestinal tract and mesentery. Surprisingly, the pathological changes observed in the pigs challenged with 10^4 ^TCID_50 _were more severe than those in the other pigs (data not shown).

**Table 3:** Average scores for the pathological changes observed in challenged Bama miniature pigs.

Groups	Heart	Liver	Spleen	Lung	Kidney	Stomach	Bladder	Lymph Nodes	Tonsils	Total
A	1.7	2.0	3.0	1.7	2.3	1.3	2.3	2.0	2.3	18.7
B	2.7	2.7	3.0	2.3	2.0	1.7	2.0	2.3	3.0	21.7
C	2.0	2.5	3.0	2.5	2.5	2.0	2.0	2.5	3.0	22.0
D	2.5	2.5	3.0	2.5	2.5	2.5	3.0	2.5	3.0	24.0
E	0.0	0.0	0.0	0.0	0.0	0.0	0.0	0.0	0.0	0.0

Extended scoring systems for clinical symptoms and pathology were used allowing the detailed characterisation of the diseases and lesions (Everett et al., 2010). Heart, liver, spleen, lung, kidney, stomach, bladder, tonsils and lymph nodes were collected from pigs that died from the infection or were euthanised at the end of the trial. Examinations followed standard operational procedures and any observed lesions were recorded. A gross pathological scoring system was used incorporating 10 parameters. Each parameter was scored from 0 (no lesion) to 3 (severe lesion).

Samples were fixed in buffered formalin, embedded in paraffin wax and stained with H&E for histopathological examination. The most common histopathological findings in the challenged and in-contact animals were mild to severe lymphoid depletion, accompanied by hyperaemia and haemorrhage. Differing degrees of lymphoid depletion were also observed in the spleen. Perivascular cuffing, due to inflammatory lymphohistiocytic infiltrates, was frequently observed in the liver and kidney. The uninfected control animals showed no obvious changes. A composite analysis using the histopathological scoring system was performed, which yielded a mean histopathological score of 15.67 in group A, 15.4 in group B, 17 in group C and 17.75 in group D (Table 4). These data indicate that the histopathological changes seen in the pigs in groups C and D were more severe than those in groups A and B. No statistically significant differences were observed between the challenged pigs and the in-contact animals.

**Table 4:** Average scores for the histopathological changes observed in challenged Bama miniature pigs.

Groups	Heart	Liver	Spleen	Lung	Kidney	Stomach	Bladder	Lymph nodes	Tonsils	Total
A	0.33	1.83	2	2	1.83	0.67	2	2.67	2.33	15.67
B	0	1.8	2.8	2.4	1.2	1.2	1.2	2.4	2.4	15.4
C	0	1.6	2.8	2.4	2.4	0.8	1.8	3	2.2	17
D	1	2	2.75	1.75	2	1.75	1.75	2.5	2.25	17.75
E	0	0	0	0	0	0	0	0	0	0

Samples were fixed in buffered formalin and embedded in paraffin wax. Sections were prepared and stained with H&E for histopathological examination. A histopathological scoring system incorporating seven parameters was used and the severity of the lesions was scored from 0 (no lesion) to 3 (severe lesion).

## Discussion

This study was aimed to develop a Bama miniature pig model of CSFV infection and to use this model to study CSFV pathogenesis. The ultimate aim is to use the model to evaluate new CSF vaccines. Developing alternative porcine models with different susceptibility to CSFV would be highly beneficial, as it would help to increase our knowledge of the molecular mechanisms involved in resistance/susceptibility to the virus. This may then, potentially, lead to breeding for enhanced CSF-resistance using marker-assisted selection, an important step toward disease control.

In this study, Bama miniature pigs were inoculated i.m. with different doses of CSFV Shimen strain, which is highly virulent in larger domestic pigs causing severe CSF [13]. The results showed that Bama miniature pigs are highly susceptible to CSFV, and infected pigs developed specific CSF clinical signs. These clinical signs were mostly consistent with those observed in experimentally infected domestic pigs. Marked CSF clinical signs appeared 2 dpi and persisted until the end of the trial. Like domestic pigs, the Bama miniature pigs showed marked fever, with the peak temperature being 42°C. There were also specific clinical signs of CSF, such as loss of appetite, lethargy, stiffness of gait and oedema. Pronounced pathological changes were also observed in the tissues and organs, including haemorrhaging in the kidney, lymph nodes and urinary bladder and necrosis in the tonsils and spleen, similar to that observed in domestic pigs infected with CSFV [13]. Unexpectedly, more severe pathological changes, higher fever frequency, and higher virus load were observed in 10^4 ^TCID_50 _group. The possible explanations may be: 1) The infected animals were housed in the same room and direct transmission between different dose groups are likely to occur; 2) The three doses studied all exceed the minimum lethal dose of the virus, thus dose effects were not revealed; 3) The differences are not statistically significantly different in most cases. To define the dose-dependent pathogenicity, smaller doses should be included in future study. Notably, the results of the present study revealed obvious differences between domestic pigs and Bama miniature pigs in terms of pathological changes in the alimentary tract. Infected miniature pigs showed severe haemorrhaging in the digestive tract, including the stomach, intestinal tract and mesentery, which are mild or absent in domestic pigs. Possibly, there may be slight differences in tissue tropism of CSFV between the two pig species, or just an occasional case. A more detailed comparative pathology study is needed to clarify the possible pathogenicity differences. No obvious clinical signs of CSF were observed in the in-contact pigs prior to death; although CSF-induced pathological changes, consistent with those observed in domestic pigs, were noted. These results suggest that Bama miniature pigs are as susceptible to CSFV as domestic pigs. More research regarding CSFV replication, proliferation, pathogenesis and host interactions (such as cytokine induction) are required, as acute overproduction of some cytokines may play a key role in pathogenesis [14].

Taking the temperature of Bama miniature pigs is very difficult as they become agitated very easily. Therefore, traditional methods (i.e., measuring rectal temperature) are not suited to these animals. This means that the recorded temperatures may not be very accurate. Thus, a more simple and accurate method for taking the temperature of these pigs, such as biochips, should be used. The use of biochips for monitoring temperatures provides practical advantages in terms of easy monitoring, which reduces disturbance to the animals and allows more frequent measurement [15]. Also, it is very important to take the temperature at consistent time points, as these animals have a marked circadian rhythm [15].

## Conclusions

Bama miniature pigs were infected with the highly virulent CSFV Shimen strain and subsequent infection/transmission was monitored establishing an experimental challenge model for CSFV. Further work is required to establish whether this Bama miniature pig model can be used to evaluate the efficacy of CSFV vaccines.

## Materials and methods

### Animals

Twenty 120-day-old SPF Bama miniature pigs (body weight, 16-25 kg) were used in the study. All pigs were clinically healthy and maintained in the animal facility at Harbin Veterinary Research Institute under standard conditions prescribed by the Institutional Guidelines. The study protocol was approved by the Institutional Animal Care and Use Committee. Each group (except the control group) was placed in an individual pen in the same room. Prior to the study, all pigs were negative for CSFV antibodies and antigens as assessed by a CSFV antibody detection kit (IDEXX, Liebefeld-bern, Switzerland) and a TaqMan real-time RT-PCR [16].

### Virus

The highly virulent CSFV Shimen strain [17] was propagated and titrated in PK-15 cells. Briefly, serial 10-fold dilutions (10^-1 ^to 10^-10^) of the virus stock were prepared in Dulbecco's modification of Eagle's medium (DMEM) and used to infect PK-15 cells (seeded in 96-well plates on the previous day at a density of 10^6 ^cells/well). Each dilution was assayed in duplicate (100 μl/well). Mock-infections (cells not exposed to virus) were set up simultaneously. After 2 h of adsorption, the virus inoculum was drawn off and DMEM containing 2% fetal bovine serum (FBS) added. The cells were then incubated at 37°C in a humidified 5% CO_2 _incubator. On Day 3 post-infection, the cells were washed twice with phosphate buffered saline (PBS) and fixed for 8 min with cold acetone. The fixed cells were then incubated with the anti-E2 monoclonal antibody (mAb) HQ06 [18] for 1 h at 37°C in a humidified chamber followed by three washes with PBS. The cells were then incubated with fluorescein isothiocyanate (FITC)-labelled goat anti-mouse IgG (Sigma-Aldrich, Missouri, USA) for 45 min at 37°C, followed by three washes with PBS. Finally, the cells were covered with 90% alkaline glycerine and examined under a fluorescence microscope (Nikon TE200, Japan).

### Animal inoculation

The pigs were randomly divided into five groups. Groups A, B and C of five pigs each were housed in three pens of one room and inoculated intramuscularly (i.m.) with a 1-ml aliquot of CSFV Shimen strain at 10^4^, 10^5 ^or 10^6 ^median tissue culture infective dose (TCID_50_), respectively. Group D of three pigs were not inoculated, but housed in the same room, serving as in-contact infection controls. Group E comprised two uninfected control animals housed in a pen of a separate room. Standard disease parameters were determined as described previously [15]. Following viral challenge, rectal temperature and clinical signs (anorexia, depression, shivering, haemorrhage, constipation and diarrhoea) were recorded on a daily basis. The tonsils, spleen, kidney, liver, lung, urinary bladder and lymph nodes were harvested from the pigs after death (from infection or after euthanasia at the end of the trial) were collected for further immunohistochemical study and evaluation of pathological changes.

### Blocking ELISA

To monitor seroconversion, serum samples were collected at 0, 3, 7, 10 and 15 dpi and tested for CSFV-specific antibodies using the IDEXX HerdChek* CSFV Antibody Test Kit (IDEXX, Liebefeld-bern, Switzerland) according to the manufacturer's instructions.

### Real-time RT-PCR

Serum samples were collected at 0, 1, 3, 7, 10 and 15 dpi to measure the level of viraemia. Tissues and organs were also collected to determine the distribution of CSFV RNA. CSFV RNA was isolated using an RNA isolation kit (Qiagen, USA). CSFV RNA was quantified by real-time RT-PCR using the TaqMan System, primers and probes specific for CSFV, and an ABI 7700 Sequence Detector (PE Applied Biosystems, USA) as described previously [16]. A standard curve was constructed using known amounts of RNA diluted in salmon sperm RNA and used to determine the viral copy number. The detection limit of the PCR assay was 41.8 RNA copies per reaction with excellent linearity (R > 0.94) over five logs of RNA content. The samples were run in triplicates. For statistical purposes data were expressed as the logarithm mean.

### Pathology and histopathology

An extended pathological and histopathological scoring system allowed detailed characterisation of pathological lesions [15]. Heart, liver, spleen, lung, kidney, stomach, urinary bladder, tonsils and lymph nodes were collected for pathological examination. Examinations followed standard operational procedures and any observed lesions were recorded. The gross pathology scoring system incorporated 10 parameters (body condition, skin and subcutis, tonsils, spleen, kidney, ileum and ileocecal valve, respiratory system, lymph nodes, urinary bladder and conjunctiva). Each parameter was scored from 0 (no lesion) to 3 (severe lesion). Samples were fixed in buffered formalin and embedded in paraffin wax. Sections (4 μm) were stained with haematoxylin and eosin (H-E) for histopathological examination. A histopathological scoring system incorporating seven parameters (tonsils, spleen, lymph nodes, liver, lung, kidney and ileocaecal valve) was also developed, and the severity of the lesions scored from 0 (no lesion) to 3 (severe lesion). Data from both scoring systems were analysed using the Kruskal-Wallis non-parametric mean comparison test [19] and differences were considered significant at *P *< 0.05.

## Authors' contributions

YS, QJ, DYT, HL, CDS and HL carried out the study. QYH and WH carried out real-time RT-PCR. SPH and ZZ performed the pathological and histopathological detection. HJQ and LDQ designed the study and wrote the paper. All authors read and approved the final manuscript.

## Competing interests

The authors declare that they have no competing interests.
